# Verification of the “Upward Variation in the Reporting Odds Ratio Scores” to Detect the Signals of Drug–Drug Interactions

**DOI:** 10.3390/pharmaceutics13101531

**Published:** 2021-09-22

**Authors:** Yoshihiro Noguchi, Shunsuke Yoshizawa, Keisuke Aoyama, Satoaki Kubo, Tomoya Tachi, Hitomi Teramachi

**Affiliations:** Laboratory of Clinical Pharmacy, Gifu Pharmaceutical University, 1-25-4, Daigakunishi, Gifu-shi 501-1196, Gifu, Japan; 185127@gifu-pu.ac.jp (S.Y.); 175003@gifu-pu.ac.jp (K.A.); 175050@gifu-pu.ac.jp (S.K.); tachi@gifu-pu.ac.jp (T.T.)

**Keywords:** spontaneous reporting systems, drug–drug interaction, reporting odds ratio (ROR), Ω shrinkage measure

## Abstract

The reporting odds ratio (ROR) is easy to calculate, and there have been several examples of its use because of its potential to speed up the detection of drug–drug interaction signals by using the “upward variation of ROR score”. However, since the validity of the detection method is unknown, this study followed previous studies to investigate the detection trend. The statistics models (the Ω shrinkage measure and the “upward variation of ROR score”) were compared using the verification dataset created from the Japanese Adverse Drug Event Report database (JADER). The drugs registered as “suspect drugs” in the verification dataset were considered as the drugs to be investigated, and the target adverse event in this study was Stevens–Johnson syndrome (SJS), as in previous studies. Of 3924 pairs that reported SJS, the number of positive signals detected by the Ω shrinkage measure and the “upward variation of ROR score” (Model 1, the Susuta Model, and Model 2) was 712, 2112, 1758, and 637, respectively. Furthermore, 1239 positive signals were detected when the Haldane–Anscombe 1/2 correction was applied to Model 2, the statistical model that showed the most conservative detection trend. This result indicated the instability of the positive signal detected in Model 2. The ROR scores based on the frequency-based statistics are easily inflated; thus, the use of the “upward variation of ROR scores” to search for drug–drug interaction signals increases the likelihood of false-positive signal detection. Consequently, the active use of the “upward variation of ROR scores” is not recommended, despite the existence of the Ω shrinkage measure, which shows a conservative detection trend.

## 1. Introduction

To ensure the proper use of drugs, it is important to understand the related adverse events. However, pre-marketing randomized clinical trials focus on establishing the safety and efficacy of a single drug, rather than investigating drug–drug interactions. Therefore, patients using other drugs along with the drug being studied in a clinical trial are excluded from an investigation. However, unlike pre-marketing studies, in actual clinical practice, multiple drugs are generally used for treatment.

Recent reports have estimated that the proportion of adverse events caused by drug–drug interactions is approximately 30% of unexpected adverse events [1]. Considering the numerous reports on polypharmacy in treatment in recent years [2,3,4,5,6], early identification of adverse events that may be caused by drug–drug interactions is an important issue that should be addressed.

Spontaneous reporting systems, which play an important role in pharmacovigilance, are a source of information for the detection of previously unknown adverse events not identified in clinical trials, including adverse events caused by drugs in post-marketing. Using spontaneous reporting systems, there are several reports of safety assessments that reflect real-world use in specific populations and clinical practice. However, the databases used in spontaneous reporting systems contain only cases of adverse events caused by the use of drugs and do not include the number of users of the drugs; therefore, the incidence of adverse events cannot be calculated. Thus, instead of incidence, the disproportionality analysis signals have been used to search for unknown adverse events [7,8,9]. The disproportionality analysis focuses on differences in the proportion of adverse event reports. If the reporting rate of the medicinal product of interest is high compared to the average reporting rate for all other medicinal products, this indicates that “the medicinal product and the adverse event have an association” [10]. For this analysis, a number of algorithms have also been reported to search for signals of drug–drug interactions [11,12]. Among these, the Ω shrinkage measure [13,14,15], proposed by Noren et al., is used by the World Health Organization Uppsala Monitoring Center (WHO-UMC), and previous studies have shown that it has the most conservative signal detection trend among signal detection methods based on frequentist statistics [16]. This study defined a conservative signal detection algorithm as one that detects few signals that are specific to that detection algorithm, and many signals that are common to other algorithms.

Surprisingly, however, there have only been few papers using the Ω shrinkage measure [17]; rather, several reports have used a detection method that extends the reporting odds ratio (ROR) score [18,19,20]. In addition, there is also a report that evaluated the ROR score of the concomitant use [21]. However, the validity of the analysis method using the ROR score has been questioned by Kuss et al., and it was discussed together with the results using another detection algorithm [22].

The ROR is an algorithm used by the Pharmaceuticals and Medical Devices Agency (PMDA) in Japan and the Pharmacovigilance Center (Lareb) in The Netherlands, which generally searches for adverse events caused by a single drug rather than drug–drug interactions [23]. 

A detection method that expands the ROR score evaluates a combination as having an increased risk of adverse events if the ROR score for the combination of drugs is higher than the ROR score for a single drug (i.e., if there is an upward variation in the ROR score). Although Susuta et al. were the first to propose a detection method that uses the “upward variation in the ROR score” as the signals for drug–drug interactions in Japan [18], the proposed cutoff values of the scores and the comparison with other methods for detecting signals of drug–drug interactions have not been sufficiently verified, and the validity of the signals detected by this analysis method is unknown. Therefore, in this study, we aimed to verify an analysis method that uses the “upward variation in the ROR scores” as the signals for drug–drug interactions, referring to similar previous studies that evaluated the detection tendency of search algorithms [16,24,25,26].

## 2. Materials and Methods

### 2.1. Data Sources

The dataset used for validation was the same dataset used in our previous study [16]. The dataset was created from the Japanese Adverse Drug Event Report database (JADER) data from the first quarter of 2004 to the fourth quarter of 2015. The JADER consists of four comma-separated values (csv) files as data tables: DEMO.csv (patient information), DRUG.csv (medicine information), HIST.csv (patient past history), and REAC.csv (AE event information). These files were linked by identification numbers, and the four csv files were combined using the identification numbers to create a database for analysis. In this study, we did not use patient information and past history, because we were only investigating the differences in detection trends between algorithms. Therefore, we removed this information when creating the verification dataset.

The Japanese authority, the PMDA, which owns these data, does not permit sharing the data directly. The latest version of the file can be accessed directly here: (http://www.info.pmda.go.jp/fukusayoudb/CsvDownload.jsp (accessed date: 10 August 2021)) (in Japanese only).

### 2.2. Targeted Drugs and Adverse Events

The drugs registered as “suspected drugs” in the verification dataset were considered as the drugs to be investigated. As in previous studies [16,18,24,25,26], the only adverse event targeted for this signal search was set to be the preferred term; that is, “Stevens-Johnson syndrome” (SJS) in the Medical Dictionary for Regulatory Activities Japanese version (MedDRA/J), which is registered in the verification dataset.

### 2.3. Statistical Models and Criteria

#### 2.3.1. Ω Shrinkage Measure

The Ω shrinkage measure that detected the most conservative signal [16] is used as the control model in this study. The score of the Ω shrinkage measure was calculated from the number of reports (*n*_111_) and its expected value (*E*_111_), as shown in Table 1, using Equations (1)–(6). The lower limit of the 95% confidence interval (CI) for the Ω shrinkage measure was Ω_025_, and a positive signal was considered to exist when Ω_025_ > 0, as previously reported [13].

(1) $$\Omega =log_{2}\frac{n_{111}+0.5}{E_{111}+0.5}\;$$

where *n*_111_ is the number of reports and *E*_111_ is the expected value.

(2) $$f_{00}=\frac{n_{001}}{n_{00+}},\;\;f_{10}=\frac{n_{101}}{n_{10+}},\;\;f_{01}=\frac{n_{011}}{n_{01+}},\;\;f_{11}=\frac{n_{111}}{n_{11+}}$$

where *n* is the number of reports shown in Table 1. For example, *n*_111_ is the number of reported target adverse event caused by *drug D*_1_ and *drug D*_2_.

(3) $$g_{11}=1-\frac{1}{\max\left(\frac{f_{00}}{1-f_{00}},\;\frac{f_{10}}{1-f_{10}}\right)+\max\left(\frac{f_{00}}{1-f_{00}},\;\frac{f_{01}}{1-f_{01}}\right)-\frac{f_{00}}{1-f_{00}}+1}$$

when *f*_10_ < *f*_00_, which denotes no risk of an adverse event caused by *drug D*_1_, the most sensible estimator *g*_11_ = max (*f*_00_, *f*_01_) is yielded and vice versa when *f*_01_ < *f*_00_.

(4) $$E_{111}=g_{11}\times n_{11+}$$

(5) $$\mathrm{Var}\left(\Omega_{0}\right)=\mathrm{Var}\left(log_{2}\frac{n_{111}}{E_{111}}\right)\approx \frac{1}{n_{111}\log{\left(2\right)}^{2}}$$

(6) $$\Omega_{025}=\Omega -\frac{\phi \left(0.975\right)}{log\left(2\right)\sqrt{n_{111}}}$$

where *ϕ* (0.975) is 97.5% of the standard normal distribution.

#### 2.3.2. Upward Variation in Reporting Odds Ratio Scores

The ROR scores were calculated using the data from the number of reports shown in Table 2 and Equations (7) and (8). The lower limit of the 95% CI for ROR was ROR_025_, and the upper limit was ROR_975_ [23].

(7) $$\mathrm{ROR}=\frac{N_{11}/N_{10}}{N_{01}/N_{00}}$$

(8) $$\mathrm{ROR}\;\left(95\%\;\mathrm{CI}\right)=e^{ln\;\left(\mathrm{ROR}\right)\pm 1.96\sqrt{\frac{1}{N_{11}}+\frac{1}{N_{10}}+\frac{1}{N_{01}}+\frac{1}{N_{00}}}}$$

where *N* is the number of reports shown in Table 2. For example, *N*_11_ is the number of reported target adverse events caused by *drug D*_1_ and *drug D*_2_.

Additionally, to calculate the ROR of *drug D*_1_ ∩ *drug D*_2_, *drug D*_1_ and *drug D*_2_, replace it as follows: *drug D*_1_ ∩ *drug D*_2_: *N*_11_ = *n*_111_, *N*_00_ = *n*_000_ + *n*_010_ + *n*_100_, *N*_10_ = *n*_110_, *N*_01_ = *n*_001_ + *n*_011_ + *n*_101_, *N*_1+_ = *n*_11+_, *N*_+1_ = *n*_++1_, *N*_0+_ = *n*_00+_ + *n*_01+_ + *n*_10+_, *N*_+0_ = *n*_++0_; *drug D*_1_: *N*_11_ = *n*_111_ + *n*_101_, *N*_00_ = *n*_000_ + *n*_010_, *N*_10_ = *n*_110_ + *n*_100_, *N*_01_ = *n*_001_ + *n*_011_, *N*_1+_ = *n*_11+_ + *n*_10+_, *N*_+1_ = *n*_++1_, *N*_0+_ = *n*_00+_ + *n*_01+_, *N*_+0_ = *n*_++0_; *drug D*_2_: *N*_11_ = *n*_111_ + *n*_011_, *N*_00_ = *n*_000_ + *n*_100_, *N*_10_ = *n*_110_ + *n*_010_, *N*_01_ = *n*_001_ + *n*_101_, *N*_1+_ = *n*_11+_ + *n*_01+_, *N*_+1_ = *n*_++1_, *N*_0+_ = *n*_00+_ + *n*_10+_, *N*_+0_ = *n*_++0_.

The signal of drug–drug interactions detected using ROR has been recently reported in Japan as “the lower limit of the 95% CI of the ROR score when *drug D*_1_ and *drug D*_2_ are used together (ROR_025_ *_drug D_*_1 ∩ *drug D*2_) > 1” and “when *drug D*_1_ and *drug D*_2_ are used together (ROR*_drug D_*_1 ∩ *drug D*2_) is greater than either the ROR score of *drug D*_1_ (ROR*_drug D_*_1_) or the ROR score of *drug D*_2_ (ROR*_drug D_*_2_), whichever is greater (Model 1).” The ROR score ratio (Model 1) is calculated using Equation (9).

(9) $$\mathrm{ROR}\;\mathrm{score}\;\mathrm{ratio}\;\left(\mathrm{Model}\;1\right)=\frac{{\mathrm{ROR}}_{drug\;D_{1}\cap \;drug\;D_{2}}}{\max\left({\mathrm{ROR}}_{drug\;D_{1}},\;{\mathrm{ROR}}_{drug\;D_{2}}\right)}$$

Susuta et al. proposed “(ROR_025_ *_drug D_*__1_ ∩ *drug D*_2__) > 1” and “ROR score ratio (Model 1) > 2” as signal detection criteria for drug–drug interactions [18]. This was evaluated as the “Susuta model.”

**Figure 1:**
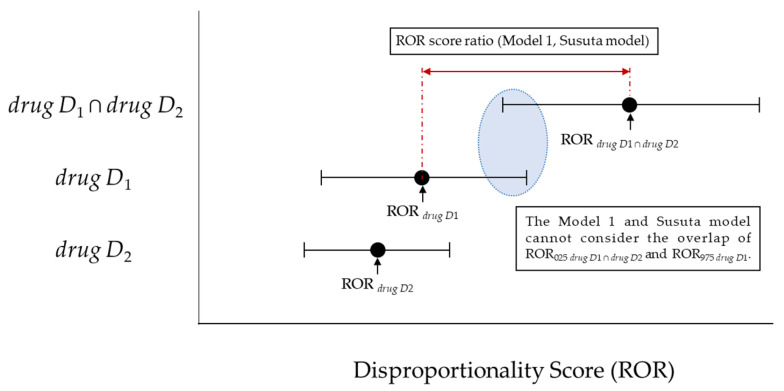
The association between the ROR score ratio (Model 1, Susuta model) and disproportionality score.

However, Model 1 and the Susuta model failed to account for the overlap between the lower 95% CI of the ROR score for *drugs D*_1_ and *drug D*_2_ together and the upper 95% CI of the ROR score for either *drug D*_1_ or *drug D*_2_, whichever is greater (Figure 1). 

**Figure 2:**
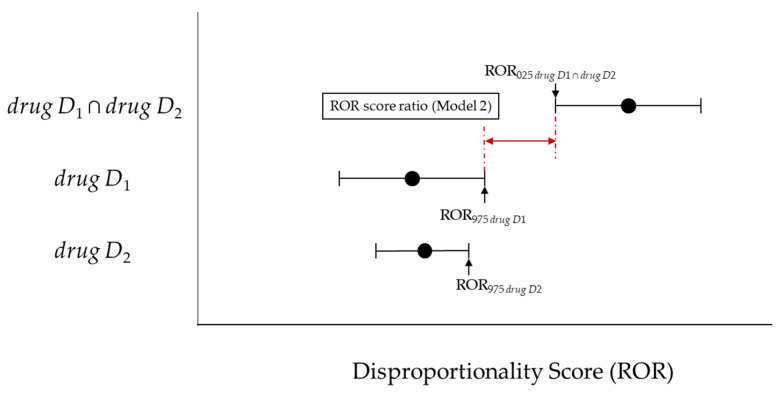
The association between the ROR score ratio (Model 2) and disproportionality score.

If such an overlap occurs, a risk signal may not be detected for concomitant use. Therefore, with reference to the interaction signal score (INTSS) [27] and concomitant signal score (CSS) [26], we used the criteria to detect if “(ROR_025_ *_drug D_*_1 ∩ *drug D*2_) > 1” and “the lower limit of the 95% CI of the ROR of the combination of *drug D*_1_ and *drug D*_2_ (ROR_025_ *_drug D_*_1 ∩ *drug D*2_) is greater than either the upper limit of the 95% CI of *drug D*_1_ (ROR_975_ *_drug D_*_1_) or the upper limit of the 95% CI of the ROR of *drug D*_2_ (ROR_975_ *_drug D_*_2_), whichever is greater (Model 2)” (Figure 2, Equation (10)).

(10) $$\mathrm{ROR}\;\mathrm{score}\;\mathrm{ratio}\;\left(\mathrm{model}\;2\right)=\frac{{\mathrm{ROR}}_{025\;drug\;D_{1}\cap \;drug\;D_{2}}}{\max\left({\mathrm{ROR}}_{975\;drug\;D_{1}},\;{\mathrm{ROR}}_{975\;drug\;D_{2}}\right)}$$

However, in the estimation of the ROR, if any one of the four cells in Table 2 is 0, the ROR will be 0 or ∞. In addition, if there is zero in the perimeter sum, the ROR cannot be defined. Furthermore, when estimating ROR when the sample size in a cell is small, as seen in a few reports and in this study, the effect of a single case in a small sample cell is very large, making the estimation unstable [28]. The Haldane–Anscombe 1/2 correction, which adds 1/2 to each cell, is known to solve this problem [29]. In this study, among the detection methods (Model 1, Susuta Model, and Model 2) that utilize the “upward variation in the ROR scores”, the statistical model that showed the most conservative detection tendency was also analyzed with the Haldane–Anscombe 1/2 correction.

### 2.4. Targeted Drugs and Adverse Events

The signal similarity of each statistical detection method was evaluated using Cohen’s kappa coefficient (*κ*), proportionate agreement for the positive rating (*P_pos_*), and proportionate agreement for the negative rating (*P_neg_*), as reported in previous studies [16,24]. Cohen’s kappa coefficient and its 95% CI were obtained from Table 3 and Equations (11)–(14) [30].

(11) $$P_{o}=\frac{N_{yy}+N_{nn}}{N_{..}}$$

(12) $$P_{e}=\frac{N_{y.}}{N_{..}}\times \frac{N_{.y}}{N_{..}}+\frac{N_{n.}}{N_{..}}\times \frac{N_{.n}}{N_{..}}$$

The number of *N_yy_*, *N_y._*, *N_y._*, *N_nn_*, *N_n._*, *N_.n_* and *N*_._ can be obtained from Table 3.

(13) $$Cohen^{\prime }s\;kappa\;coefficient\;\left(\kappa \right)=\frac{P_{o}-P_{e}}{1-P_{e}}$$

(14) $$95\%\;CI\;of\;kappa\;coefficient=\kappa \pm 1.96\sqrt{\frac{P_{o}\times \left(1-P_{o}\right)}{N_{..}\times {\left(1-P_{e}\right)}^{2}}}$$

The *P_pos_* and *P_neg_* can be obtained from Equations (15) and (16) and Table 3 [27].

(15) $$P_{pos}=\frac{2N_{yy}}{N_{y.}+N_{.y}}$$

(16) $$P_{neg}=\frac{2N_{nn}}{N_{n.}+N_{.n}}$$

However, the number of *N_yy_*, *N_y._*, *N_y._*, *N_nn_*, *N_n._* and *N_.n_* can be obtained from Table 3.

## 3. Results

Table 4 shows the number of reports (*n*_111_) and the number of signals detected for each of the Ω shrinkage measure, Model 1, the Susuta model, Model 2, and Model 2 (Haldane–Anscombe 1/2 correction).

Of the 374,327 cases used in this study, there were 3924 *drug D*_1_–*drug D*_2_-SJS, wherein the number of positive signals was 712 for the Ω shrinkage measure [16]; 2,112 for Model 1; 1758 for the Susuta model; and 637 for Model 2. In Model 1, the Susuta Model, and Model 2, 1239 positive signals were detected when Haldane–Anscombe 1/2 correction was applied to Model 2, which, consequently, was the statistical model that showed the most conservative detection trend.

Table 5 shows the *κ*, *P_pos_*, and *P_neg_* between the Ω shrinkage measure and the detection methods of Model 1, the Susuta model, Model 2, and Model 2 (Haldane–Anscombe 1/2 correction).

The detection method with the most similar detection tendency as the Ω shrinkage measure was Model 2 (*κ*: 0.495; 95% CI: 0.475–0.514; *P_pos_*: 0.581; *P_neg_*: 0.913), followed by the Susuta model (*κ*: 0.152; 95% CI: 0.135–0.168; *P_pos_*: 0.371; *P_neg_*: 0.711) and Model 1 (*κ*: 0.074; 95% CI: 0.058–0.089; *P_pos_*: 0.325; *P_neg_*: 0.621).

However, the similarity of the detection trend of Model 2 decreased when the Haldane–Anscombe 1/2 correction was applied (*κ*: 0.476; 95% CI: 0.459–0.492; *P_pos_*: 0.597; *P_neg_*: 0.867).

**Figure 3:**
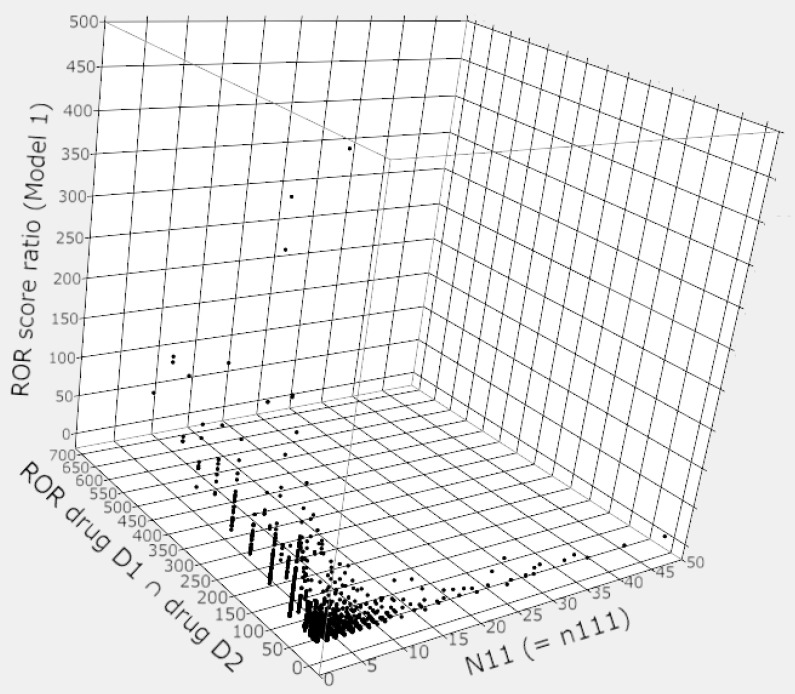
Relationship between the number of reports (*N*_11_ [= *n*_111_]), ROR*_drug D_*_1 ∩ *drug D*2_, and ROR score ratio (Model 1).

Figure 3 shows the relationship between the number of reports (*n*_111_), the ROR score (ROR*_drug D_*_1 ∩ *drug D*2_), and the ROR score ratio when two *drug D*_1_ and *drug D*_2_ were used in combination.

In cases where the number of reports (*N*_11_ = [*n*_111_]) was less than 10, there were many cases where the ROR*_drug D_*_1 ∩ *drug D*2_ exceeded 50, and the maximum score was over 700. The ROR score ratio (Model 1) was also inflated by the inflation of the ROR*_drug D_*_1 ∩ *drug D*2_, the maximum score of ROR score ratio (Model 1) was over 450 (Figure 3).

However, some of the combinations with the very high ROR*_drug D_*_1 ∩ *drug D*2_ or the ROR score ratios do not meet the detection criteria for the Ω shrinkage measure, e.g., *N*_11_: 11, ROR*_drug D_*_1 ∩ *drug D*2_: 57.45, ROR score ratio (Model 1): 3.43, Ω_025_: −0.18.

## 4. Discussion

In this study, we examined a method of using the “upward variation in the ROR score” as a signal of drug–drug interactions. Strictly, detection trends should be compared for all adverse events registered in the database. Unfortunately, even with fast and powerful computers, calculating signal scores for all combinations of multiple drugs and adverse events can be expected to take an enormous amount of time; thus, targeting all combinations was not a realistic research method. Therefore, in this study, as in a previous study [16,18,24], the target adverse event was SJS.

Of the 3924 combinations of *drug D*_1_–*drug D*_2_-SJS, 2112 positive signals were detected in Model 1, 1758 in the Susuta model, and 637 in Model 2. All of the combinations detected in Model 2 also had a positive signal detected in Model 1. Additionally, of the 637 combinations detected in Model 2, 636 pairs also had a positive signal detected in the Susuta model (Table 4). 

The Ω shrinkage measure detected 712 positive signals [16]. The detection method with the most similar detection tendency as the Ω shrinkage measure was Model 2 (Table 5), with 392 positive signals in common. However, among the detection methods that utilize the “upward variation in ROR score”, even in Model 2, which showed the most conservative detection tendency, even one of the four cells in Table 2 became a 0 cell, and there were 1160 pairs in which the ROR score ratio could not be calculated (Table 4). The problem of negative signals due to the inability to calculate this score was solved by Haldane–Anscombe 1/2 correction; however, the number of positive signals increased by 602 from that before the correction, for a total of 1239 (Table 4). This result indicated the instability of the positive signal detected in Model 2.

Furthermore, even Model 2, which had the highest similarity to the Ω shrinkage measure in this study, had a similarity lower than that between the Ω shrinkage measure and the Chi-square model [31] in a previous study using the same verification database and the same targeted adverse event [16].

The signals obtained from JADER, the database used in this study, require verification to confirm that they are true adverse events, and true data are needed to evaluate the validity of the detection results. However, it is not possible to prepare true data, including the data of “unknown” adverse events.

In the verification of a signal detection method for a single drug, Szarfman et al. reported [32] that the information in the medical package inserts was set as the “true” data and evaluated using receiver operating characteristic (ROC) curves with different cutoff values for signal scores [32]. However, as Watanabe et al. pointed out, it is unclear whether the information in the package insert is the only “true” data, and this verification method has its limitations [33].

In fact, considering that signal detection methods are designed to search for “unknown” adverse events, it is not appropriate to evaluate the performance of detection methods using only “known” information. Thus, following the combinations of drug–drug interactions described in the medical package insert, as in Kubota et al. and our previous studies [16,24], we only compared the detection trends of each statistical model in this study. This study is not affected by patient background, as it only shows the difference between the calculated results of each statistical model and the interpretation of the signal scores. This is the same as previous studies [16,18,24]. 

However, this limitation of not being able to provide “true” data makes it difficult to determine whether the signal detection results from the detection method using the “upward variation in ROR score” are overestimated or whether the signal detection results from the Ω shrinkage measure are underestimated.

This study was conducted under limited conditions and may require further investigation using simulation data. However, unlike the Bayesian confidence neural network (BCPNN) [34] and the empirical Bayes geometric mean (EBGM) [35] based on Bayesian statistical methods, the ROR based on frequency-based statistical methods is prone to signal score inflation when the number of reports is small [36], leading to unstable detection results [37]. 

In general, the number of reports for the combination of two drugs (*drug D*_1_ ∩ *drug D*_2_) will be less than the number of reports for single drug use (*drug D*_1_ or *drug D*_2_); therefore, the ROR score (ROR*_drug D_*_1 ∩ *drug D*2_) is likely to be inflated. In fact, in this study, there were many cases of inflation of ROR*_drug D_*_1 ∩ *drug D*2_, as shown in Figure 3. The ROR*_drug D_*_1 ∩ *drug D*2_ is a numerator in the equation of Model 1 and the Susuta model, and such signal score inflation may make it easier to detect false-positive signals of drug–drug interactions.

Further, it is known that the 95% CIs are wider when the number of reports is small, and in this study, the method that did not take into account the overlap between the 95% CI of the signal score for the combination of two drugs (*drug D*_1_ ∩ *drug D*_2_) and the 95% CI of the signal score for single drug (*drug D*_1_ or *drug D*_2_) use (Model 1, Susuta model) resulted in a higher likelihood of detecting false-positive signals than Model 2, the method that took into account the overlap.

The results indicated that when the number of reports was small, as in the case of drug–drug interactions, it was important to consider the overlap between the 95% CI of the signal score for the combination of two drugs (*drug D*_1_ ∩ *drug D*_2_) and the 95% CI of the signal score for single drug use (*drug D*_1_ or *drug D*_2_) when calculating the ROR score ratio.

The spontaneous reporting systems, which are used for disproportionality analysis, consist only of spontaneously “reported” cases and, naturally, do not include those that occur but are not reported. Additionally, reports are known to contain a variety of biases [38,39,40]. Therefore, the calculated signal score is also affected by the biases. Spontaneous reports often lack information on concomitant medications, leading to underestimation of the drug–drug interaction signal score in some cases. Thus, the use of any statistical analysis method cannot overcome the inherent qualitative and quantitative limitations of spontaneous reporting systems [37].

The ROR is simple to calculate, and utilizing the “upward variation in the ROR score” may speed up the detection of drug–drug interaction signals. However, even Model 2, which showed the most conservative detection tendency among the detection methods utilizing the “upward variation in the ROR score”, did not show stable signal detection results due to the small number of reports, making it difficult to deny the overestimation of positive signals of drug–drug interactions, as in Model 1 and the Susuta model.

It is known that there are many attentive points when analyzing a spontaneous reporting database, and various analysis algorithms have been proposed [10]. Considering the history of the development of the BCPNN and the EBGM based on Bayesian statistical models [37] to avoid signal score inflation that detects a large number of false-positive signals in the detection of single drug signals, even though there is not only the Ω shrinkage measure used in WHO-UMC, but also several alternative detection methods for signal detection of drug–drug interactions [10,11], there is no reason to actively recommend the use of “upward variation in the ROR score”, which is more likely to detect false-positive signals.

## 5. Conclusions

Recently, many patients have been concomitantly using drugs, and in order to use drugs appropriately, it is necessary to screen not only for single drugs but also for safety signals such as drug–drug interactions. In this study, Model 2, which corrects the problems contained in Model 1 by referring to INTSS and CSS, was also examined. However, the ROR scores based on the frequency-based statistics are easily inflated; thus, the use of the “upward variation of ROR scores” in either statistic model to search for drug–drug interaction signals increases the likelihood of false-positive signal detection. Although, some researchers have used “upward variation of ROR scores” (the active use of this algorithm is not recommended), because of the existence of the Ω shrinkage measure, which shows a conservative detection trend. In order to reduce false-positive signals, the selection of appropriate detection algorithms is desired.

## Figures and Tables

**Table 1:** The 4 × 2 contingency table for signal detection.

		**Target AE**	**Other AEs**	**Total**
	
***drug D*_1_ and *drug D*_2_**		*n*_111_	*n*_110_	*n*_11+_
***drug D*_1_ without *drug D*_2_**		*n*_101_	*n*_100_	*n*_10+_
***drug D*_2_ without *drug D*_1_**		*n*_011_	*n*_010_	*n*_01+_
**Neither *drug D*_1_ or *drug D*_2_**		*n*_001_	*n*_000_	*n*_00+_
**Total**		*n*_++1_	*n*_++0_	*n*_+++_

AE: adverse event, *n*: the number of reports (e.g., *n*_111_: the number of target drugs (*drug D*_1_ and *drug D*_2_) induced AE, *n*_+++_: the number of all reports).

**Table 2:** The 2 × 2 contingency table for signal detection.

		**Target AE**	**Other AEs**	**Total**
	
**Target drug (s)**		*N*_11_	*N*_12_	*N*_1+_
**Other drugs**		*N*_21_	*N*_22_	*N*_2+_
**Total**		*N*_+1_	*N*_+2_	*N*_++_

AE: adverse event, *N*: the number of reports (e.g., *N*_11_: the number of target drug-induced AE, *N*_++_: the number of all reports).

**Table 3:** Agreement between the Ω shrinkage measure and target model.

		**Ω Shrinkage Measure**		**Total**
		**Yes**	**No**	
**Target model**	**Yes**	*N_yy_*	*N_yn_*	*N_y._*
	**No**	*N_ny_*	*N_nn_*	*N_n._*
**Total**		*N_.y_*	*N_.n_*	*N_.._*

Target model: model 1; model 2; Susuta model; Yes: signal detection; No: not signal detection.

**Table 4:** The *drug D*_1_–*drug D*_2_-SJS combinations detected as a potential signal in five frequency statistical models.

Statistical Models	Signal (Y/N)	Number (%) of Combinations					
		*n*_111_ < 3	*n*_111_ = 3	*n*_111_ = 4	*n*_111_ = 5	*n*_111_ > 5	Total
Model 1	Y	1363 (47.8)	243 (65.3)	159 (66.8)	110 (80.3)	237 (72.3)	2112 (53.8)
	N	525 (18.4)	48 (12.9)	23 (9.7)	14 (10.2)	42 (12.8)	652 (16.6)
	N (no criterion)	961 (33.7)	81 (21.8)	56 (23.5)	13 (9.5)	49 (14.9)	1160 (29.6)
Susuta model	Y	1142 (40.1)	207 (55.6)	136 (57.1)	97 (70.8)	176 (53.7)	1758 (44.8)
	N	746 (26.2)	84 (22.6)	46 (19.3)	27 (19.7)	103 (31.4)	1006 (25.6)
	N (no criterion)	961 (33.7)	81 (21.8)	56 (23.5)	13 (9.5)	49 (14.9)	1160 (29.6)
Model 2	Y	239 (8.4)	106 (28.5)	84 (35.3)	74 (54.0)	134 (40.9)	637 (16.2)
	N	1649 (57.9)	185 (49.7)	98 (41.2)	50 (36.5)	145 (44.2)	2127 (54.2)
	N (no criterion)	961 (33.7)	81 (21.8)	56 (23.5)	13 (9.5)	49 (14.9)	1160 (29.6)
Corrected Model 2	Y	678 (23.8)	178 (47.8)	118 (49.6)	92 (67.2)	173 (52.7)	1239 (31.6)
	N	2171 (76.2)	194 (52.2)	120 (50.4)	45 (32.8)	155 (47.3)	2685 (68.4)
	N (no criterion)	0 (0.0)	0 (0.0)	0 (0.0)	0 (0.0)	0 (0.0)	0 (0.0)
Total		2849	372	238	137	328	3924

SJS: Stevens–Johnson syndrome; *n*_111_: the number of reported target adverse event caused by *drug D*_1_ and *drug D*_2_ (see Table 1).

**Table 5:** The Cohen’s kappa coefficient and proportionate agreement for positive rating (*P_pos_*) and that for negative rating (*P_neg_*) between Ω Shrinkage measure and four frequency statistical models.

		Model 1	Susuta Model	Model 2	Corrected Model 2 *
	
Ω Shrinkage measure		*κ*: 0.074 95% CI: 0.058–0.089 *P_pos_*: 0.325 *P_neg_*: 0.621	*κ*: 0.152 95% CI: 0.135–0.168 *P_pos_*: 0.371 *P_neg_*: 0.711	*κ*: 0.495 95% CI: 0.475–0.514 *P_pos_*: 0.581 *P_neg_*: 0.913	*κ*: 0.479 95% CI: 0.459–0.493 *P_pos_*: 0.597 *P_neg_*: 0.867

*κ*: Cohen’s kappa coefficient; *P_pos_*: proportionate agreement for positive rating; *P_neg_*: proportionate agreement for negative rating, *: Model 2 with the Haldane–Anscombe 1/2 correction.

## Data Availability

This study was used the dataset from the first quarter of 2004 to the fourth quarter of 2015 from the Japanese Adverse Drug Event Report database (JADER). However, the Japanese authority, the Pharmaceuticals and Medical Devices Agency (PMDA), which owns this data, does not permit sharing the data directly. Therefore, it can be accessed directly here: (http://www.info.pmda.go.jp/fukusayoudb/CsvDownload.jsp (accessed date: 10 August 2021)) (in Japanese only).
